# Childhood bronchial mucoepidermoid tumors: A case report and literature review

**DOI:** 10.3892/ol.2013.1529

**Published:** 2013-08-16

**Authors:** XIAOZHE QIAN, ZHIYONG SUN, WENBIAO PAN, QING YE, JUN TANG, ZIANG CAO

**Affiliations:** Department of General Thoracic Surgery, Renji Hospital, Shanghai Jiaotong University School of Medicine, Shanghai 200127, P.R. China

**Keywords:** pediatric mucoepidermoid carcinoma, children, lung cancer

## Abstract

Primary pulmonary neoplasms rarely occur in children, but the majority of those that do are malignant. Mucoepidermoid carcinoma (MEC) represents ~10% of all primary pulmonary malignant tumors. However, MEC is not usually considered in the clinical differential diagnosis in pediatric practice. The present study presents the case of a seven-year-old female with a one-year history of recurrent hemoptysis. Computerized tomography (CT) scans revealed a tumor originating in the right lower lobe bronchus. The patient did not receive any radiation and chemotherapy following a lobectomy on the right lower lung. The tumor was histopathologically determined to be an MEC of the tracheobronchial tree. Subsequent to a six-year follow-up, the MEC was undetectable in this patient, according to the clinical and radiological evidence. The literature with regard to pediatric MEC is also reviewed in this study.

## Introduction

Mucoepidermoid carcinoma (MEC) is the most common type of salivary gland malignancy in adults. It is also found in other areas, including bronchi, lacrimal sac and thyroid. MEC is not common in the lungs, particularly in children, accounting for only 0.1–0.2% of primary lung cancers. Generally, pulmonary MEC presents as a painless, slow-growing mass that is firm or hard, which may block bronchial tubes and cause obstructive pneumonia. Although the tumor is not encapsulated, the tumor usually has a low malignant potential (1–3). MEC is characterized by squamous cells, mucus-secreting cells and intermediate cells, and is histologically diagnosed by Alcian blue and periodic acid-Schiff staining. The present study describes the case of a seven-year-old female with MEC. Following the appropriate treatment, the patient appeared normal during the six-year follow-up period. Written informed consent was obtained from the patient.

## Case report

**Figure 1:**
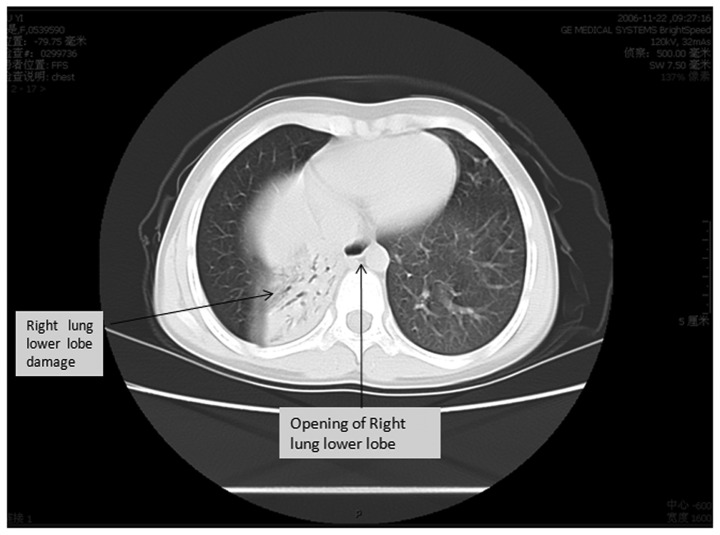
Computed tomography (CT) scan revealing atelectasis of the right lower lobe (arrow) associated with local partial pulmonary tissue collapse and a consolidation of the right middle and lower lobes.

A seven-year-old female presented with a one-year history of recurrent hemoptysis with no evident cause. A sudden onset of hemoptysis had compelled the patient to seek treatment at the Renji Hospital (Shanghai, China). An initial diagnosis indicated that the patient was apyrexial, experiencing bronchiectasis and producing diminished breath sounds in the right inferior zone. Upon further examination, a computed tomography (CT) scan revealed atelectasis of the right lower lobe associated with local partial pulmonary tissue collapse and a consolidation of the right middle and lower lobes (Fig. 1). The remainder of the bronchial tree appeared normal. One year prior to admittance to the Renji Hospital, the patient was admitted to the emergency department of a local grade three and first-class hospital due to coughing and hemoptysis. The emergency doctors prescribed antibiotics and hemostatics, which partially relieved the symptoms. However, the disease recurred six months later and the patient was administered the same treatment, which provided some relief. One year after the first hospitalization, the hemoptysis volume had doubled and the patient was transferred to the Renji Hospital. A bronchoscopy was performed at a hospital in Hangzhou (Zhejiang, China). However, the bronchoscopy may have caused airway bleeding, resulting in an obscured visual field and immediate termination of the procedure. In addition, the patient had no significant individual or family history of pulmonary disease. However, the patient’s grandmother had succumbed due to colorectal carcinoma a few years prior to this incidence.

Based on the previous diagnosis and information, the patient underwent surgery in the Renji Hospital. During surgery, congestion-like changes to the bronchial tissues were discovered in the lower lobe of the right lung, and several bleeding sites were identified in the airway. A granulomatous mass was located at the site of the middle lobar bronchial opening. In addition, a right lower and middle lobectomy was performed, followed by lymph node dissection.

**Figure 2:**
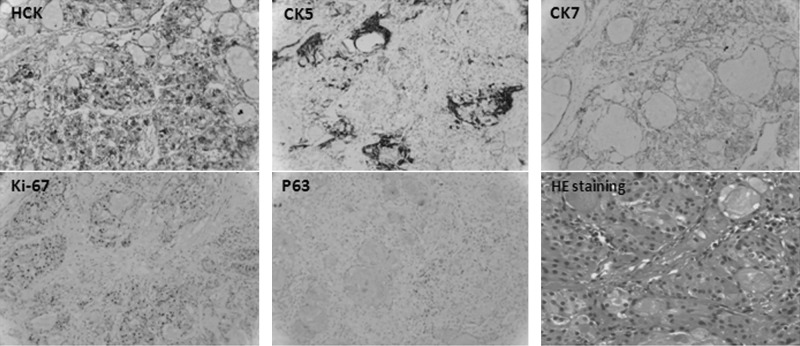
Histochemical staining of various cytokeratins, including high-molecular weight cytokeratin (HCK), CK5 and CK7. Ki-67 and p63 are also shown in the lower row. Hematoxylin and eosin (HE) staining showing a typical mucoepidermoid carcinoma.

**Figure 3:**
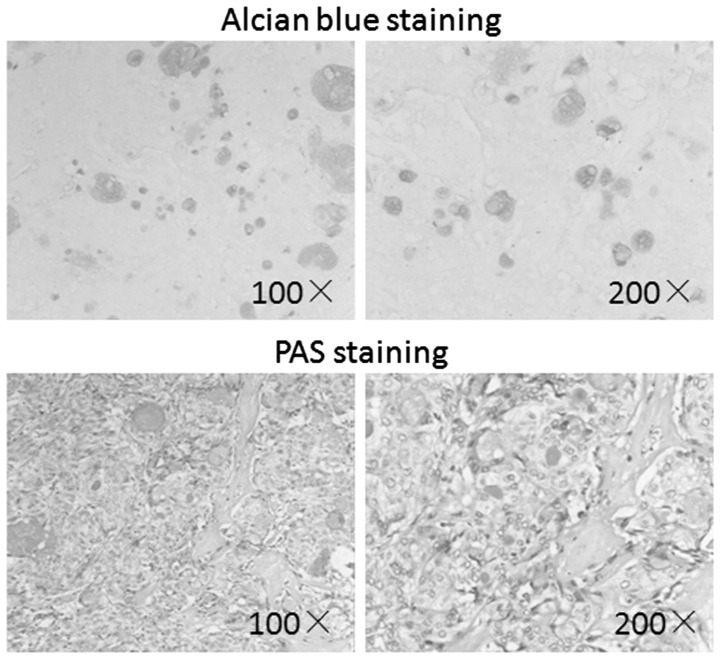
Alcian blue (AB) staining and periodic acid-Schiff (PAS) staining revealing numerous mucin deposits in the tissues. The AB and PAS staining images are shown in two magnifications (×100 and ×200).

The histology of the endobronchial specimen revealed a grade II MEC with clean resection margins. The carcinoma was 2.5×1.5×1.5 cm^3^ in size. Although histopathology revealed that the majority of the carcinoma was localized within the epidermis of the bronchus, certain sections had invaded the bronchial wall. An immunohistochemical examination revealed that the carcinoma cells were positive for high-molecular weight cytokeratin (HCK), also known as 34βE12 (+++), CK5 (++), CK7 (+++), Ki67 (+) and p63 (+; Fig. 2) and negative for p27, TTF-1, EGFR, p53, CD10 and p63 (data not shown), where + is trace/negative, ++ is weakly positive and +++ is strongly positive. Alcian blue (AB) and periodic acid-Schiff (PAS) staining were used to detect the presence and distribution of the acidic and neutral carbohydrates, respectively. AB and PAS staining was positive in the MEC specimen (Fig. 3). Based on the evidence, the patient was diagnosed with stage Ib (T2aN0M0) non-small cell lung cancer, according to the staging guidelines (4). A pathological analysis also indicated that the MEC in this patient did not differ from that observed in adult patients.

Following the surgery, the seven-year-old patient made an uneventful post-operative recovery, and the follow-up examination six years later did not reveal any clinical or radiological evidence of disease recurrence.

## Discussion

MEC is a malignant glandular epithelial neoplasm with typical characteristics, including mucous and epidermoid cells and oncotic features (5). MEC commonly occurs in the salivary glands, but is also observed in other organs, including the breast, pancreas, thyroid gland, trachea and bronchus (6). MEC of the trachea and bronchi is rare, accounting for 0.1–0.5% of all lung carcinomas (7,8). MEC is one of the most common childhood primary malignant tumors, representing ~10% of malignant lung tumors in children (9). Although the age range of patients with MEC in the lung is extensive, young patients, particularly children, are not commonly observed (7). Rapidis *et al*(10) reported an age range of 29–86 years in a cohort of 18 patients, while Ozawa *et al*(11) reported age ranges of 22–86 years and 33–70 years in cohorts of 43 patients and 30 patients, respectively (5). Cases involving patients of <10 years old are rare. Due to the rarity of this pulmonary carcinoma in children, MEC is usually not the first consideration when a child presents with recurrent atelectasis or respiratory tract infection. As a result, the diagnosis may be delayed (9). Symptoms are often present for up to 20 months prior to this delayed diagnosis (mean, 11 months) (12). In the present case, the diagnosis was delayed by one year.

The common symptoms and signs of MEC of the bronchus are coughing, hemoptysis, bronchitis, wheezing, fever, chest pain and clubbing of the fingers (13). Children with bronchial tumors are more likely to present symptoms that are more specific than their adult counterparts. Tsuchiya *et al*(14) and Dinopoulos *et al*(13) summarized the symptoms of MEC in child patients and concluded that coughing, hemoptysis, fever and recurrent pneumonia were the most common signs. Clubbing, wheezing and recurrent colds have also been observed (14). Bronchial MEC usually occurs in the main or lobar bronchi as a soft, vascular polypoid mass that induces bronchial obstruction, recurrent pneumonias, coughing and/or hemoptysis (15). With growth, the tumor initially partially and eventually completely, obstructs the bronchus, thus interfering with distal ventilation. The patient develops dyspnoea, wheezing and cough. Further obstruction results in pneumonia and atelectasis, which if unresolved, may result in bronchiectasis (16). In the present study, the initial clinical signs were a one-year history of recurrent hemoptysis and coughing. Antibiotics and hemostatics relieved the symptoms of the disease, which was also confusing. If recurrent hemoptysis, coughing and pneumonia or pneumonia-like symptoms occur in child patients, MEC should be considered during the diagnosis. Tests, including a radiographical diagnosis, plain radiography, bronchography and CT should be performed to confirm the diagnosis (17).

Since CT is the most sensitive technique for detecting and characterizing parenchymal disease, it has become the procedure of choice for further investigation. CT may be useful in assessing the extent of the disease. Familiarity with the appearance of these lesions on CT may assure an accurate diagnosis and optimize the management of the condition of the patient (18). In the present study, only CT was performed, which revealed right lower lobe atelectasis associated with local partial pulmonary tissue collapse and a consolidation of the right middle and lower lobes (Fig. 1). Although CT detected MEC in the present study, the radiographic differential diagnosis is not useful in evaluating MEC with small lesions (19). Several direct approaches, including fiberoptic bronchoscopy and ^18^F-fluorodeoxyglucose positron emission tomography (FDG-PET)/CT imaging (20–22), may avoid the problems that are associated with radiographical techniques. However, in the present case, the young patient experienced hemoptysis accompanied by airway bleeding, and a bronchoscopy may have caused massive hemorrhage. Therefore, bronchoscopy should be used more carefully or stopped when severe airway bleeding occurs. These shortcomings highlight the urgent requirement for a new method for the diagnosis of pediatric MEC that does not cause airway bleeding.

Generally, when an endobronchial mass is present in a child, the clinical consideration typically includes the aspiration of exogenous objects, respiratory tract papillomatosis, an inflammatory pseudotumor and, rarely, a metastatic or primary tumor. Whether the mass is benign or malignant may be determined based on the clinical signs or symptoms alone. The total removal of the lesion, with the sacrifice of as little normal lung tissue as possible, is required. When technically possible, a sleeve resection of the involved bronchus is recommended. However, in the majority of cases, the location of the lesions requires a lobectomy for complete removal (23,24). In the present study, an appropriate surgical procedure was performed. It is well known that planning a precise follow-up procedure depends on the disease characteristics, including the pathological type or the accurate location of the lesion. Therefore, in the present study, a right lower and right middle lobectomy was performed. Following this, a lymph node dissection was performed.

The pathological diagnosis is indispensable (25). Histological analysis is the last and the most significant method for staging MEC. Histological staining is useful for determining the properties and grades of specimens (1,25). Bronchial MEC usually forms neoplastic excretory duct reserve cells, which are histologically similar to MEC of the salivary glands (13,26). Several cytokeratins, including AE1, CK7, CK5/6, CK7 and CK8, as well as PCNA, Ki-67, p63, p53, CD10 and p27, are common markers that are used to identify MEC (1,25,27). In addition, certain other types of staining are also required, including hematoxylin and eosin (HE), AB and PAS. In the present study histological staining was performed for protein 34βE12 (+++), CK7 (+++), CK8 (+), Ki67 (+), p27, TTF-1, EGFR, p53 CD10 and p63 (Fig. 2). PAS and AB staining was also performed (Fig. 3). The histological and pathological staining revealed that the MEC was stage Ib and of a low/intermediate grade, which is consistent with previous results reported in the literature (28).

In children, mucoepidermoid tumors should be considered potentially malignant. Since these tumors are relatively slow growing, a prompt diagnosis and early surgical treatment offer the best chance of a cure in this type of patient (29). Thoracotomy is the main treatment for the total excision of the lesion. Although extensive local invasion through the tracheobronchial wall may occur, this cancer rarely exhibits distant metastasis. This tendency results in an excellent long-term outcome following surgical excision with clear margins, even without adjuvant chemotherapy or radiation therapy (30).

In the present study, the patient was normal with no clinical or radiological evidence of disease recurrence during a six-year follow-up period following the surgery and lymph node dissection.

In summary, childhood MEC is relatively rare and exhibits symptoms that are similar to those of other lung diseases, which may therefore delay a precise diagnosis. A timely examination and appropriate treatment may cure or at least markedly improve the outcome of children with MEC. More attention should be paid to increasing environmental pollution, as it may be a cause of the increase in childhood lung cancer (31,32).
